# Enhanced separation of long-term memory from short-term memory on top of LSTM: Neural network-based stock index forecasting

**DOI:** 10.1371/journal.pone.0322737

**Published:** 2025-06-02

**Authors:** Hongfei Xiao

**Affiliations:** 1 Postdoctoral Research Station, Shenwan Hongyuan Securities Co., Ltd., Shanghai, China; 2 Faculty of Finance, City University of Macau, Macao, China; 3 School of Economics, Jinan University, Guangzhou, China; University of Queensland, AUSTRALIA

## Abstract

LSTM (Long Short-Term Memory Network) is currently extensively utilized for forecasting financial time series, primarily due to its distinct advantages in separating the long-term from the short-term memory information within a sequence. However, the experimental results presented in this paper indicate that LSTM may struggle to clearly differentiate between these two types of information. To overcome this limitation, we propose the ARMA-RNN-LSTM Hybrid Model, aimed at enhancing the separation between the long-term and short-term memory information on top of LSTM framework. The experiment in this paper is inspired by an observation: when LSTMs and RNNs are respectively used to forecast the same time series that contains only short-term memory information, LSTMs exhibit significantly lower forecasting accuracy than RNNs, and we attributed this to LSTMs potentially misclassifying some short-term memory information as long-term during forecasting process. Further, we speculate that this confusion might also arise when LSTMs are used to forecast the time series containing both the long-term and short-term memory information. To verify the aforementioned hypothesis and improve the forecasting accuracy for financial time series, this paper combines RNNs with LSTMs, proposing a method of ARMA-RNN-LSTM Hybrid Modelling, and conducts an experiment with stock index prices. Eventually, the experiment results show that the ARMA-RNN-LSTM Hybrid Model outperforms standalone RNNs and LSTMs in forecasting stock index series containing both long-term and short-term memory information, confirming that the ARMA-RNN-LSTM Hybrid Model has effectively enhanced the separation between the long-term and short-term memory information within sequence. This hybrid modelling approach has innovatively addressed the issue of the confusion between the long-term and the short-term memory information in a sequence during LSTM’s forecasting process, improving the accuracy of forecasting financial time series, and demonstrates that neural network’s forecasting errors is a area worth to explore in the future.

## 1. Introduction

The autoregressive model (AR) enjoyed widespread adoption in time series forecasting, especially in forecasting stock prices [1], until the present of more advanced models like ARIMA, VAR, GARCH, and their derivatives [2–4].

While time series models have been widely used for decades to forecast stock prices [5, 6], they have consistently faced some limitations. Specifically, these models tend to focus exclusively on the short-term impact of historical prices on future prices while neglecting the potential long-term memory in price sequences. For instance, when applying the ARIMA (*p, d, q*) model to forecast stock prices, generally, neither the value of p nor q is larger than 5 [7].

In recent decades, neural networks, particularly RNNs, have gained widespread use in time series forecasting. However, traditional RNNs struggle to capture long-term memory information due to vanishing gradient problem. Consequently, the question of employing a model to process the time series with the long-term memory remained unsolved until the present of the Long Short-Term Memory Network (LSTMs) [8]. LSTMs can regulate the retention and discarding of information through their gating mechanisms, which include forget gates, input gates, and output gates. This functionality enables the LSTM model to process information over various time durations and effectively separating the long-term from short-term memory by preserving essential information while discarding irrelevant details.

When using LSTMs for time series forecasting, their key potential lies in separating the long-term memory information from the short-term, as any confusion between these two types of information can significantly diminish forecasting accuracy [9]. However, it remains challenges to evaluate the effectiveness of the information separating process within an LSTM, due to the fact that the mathematical mechanism underlying the LSTM’s forecasting has not yet been uncovered until present [10]. Therefore, we come up with a supposition of using a specific model to separate the long-term memory and the short-term memory information ‘in vitro’ of the LSTM model, by which to improve the time series forecasting accuracy. To validate this supposition, we accomplish the following work.

Firstly, proposing an approach of ARMA-RNN-LSTM hybrid modeling. This approach leverages RNNs and LSTMs to separately capture the short-term and long-term memory information within the same sequence: an RNN model is utilized to capture short-term memory information, while an LSTM model is employed to capture long-term memory information. Subsequently, the outputs generated by both the RNN and LSTM are integrated and fed into another LSTM model for forecasting. This hybrid approach enhances the forecasting capabilities by leveraging the strengths of both RNNs and LSTMs.

Secondly, conducting an experimental test. This test selects three representative groups of Chinese stock indices as sample sequences: the Shanghai Composite Index (SSE), the Shenzhen Composite Index (SZSE), and the Hang Seng Composite Index (HSI). By estimating the Hurst exponent for each of these indices, we evaluate their long-term memory characteristics. Our findings indicate that both the SZSE and the HSI demonstrate significant long-term memory, whereas the SSE does not exhibit this characteristic.

Further, the experimental results reveal that, for the SZSE and the HSI, which exhibit both the long-term and the short-term memory, the ARMA-RNN-LSTM hybrid model achieves significantly higher forecasting accuracy in comparison to the RNN model and the LSTM model; Conversely, for the SSE, which lacks the long-term memory, the RNN model achieves the highest forecasting accuracy compared to other models. This suggests that the ARMA-RNN-LSTM hybrid model is particularly advantageous for processing the time series with both long-term and short-term memory information, outperforming the LSTM model. To sum up, these findings confirm the feasibility of the ARMA-RNN-LSTM hybrid modeling.

This paper proposes an innovative approach to separate the long-term memory information from the short-term within a sequence, improving the financial time series forecasting accuracy and offering valuable insights for future researches in related fields, and providing practical guidance for stock market investors.

## 2. Literature review

This paper employs the Hurst Exponent to assess whether the time series being forecasted exhibits long-term memory characteristics. Based on this assessment, it proposes a forecasting method referred to as ARMA-RNN-LSTM hybrid modeling in this paper to forecast the sequences with long-term memory properties. Within the framework of ARMA-RNN-LSTM hybrid modeling, various models are utilized, including the AR model, the LSTM model, and the RNN model.

### 2.1. Hurst exponent

In the 1940s, H.E. Hurst, a British hydrology expert, studied the water levels of the Nile River reservoir as part of a dam project of his, and discovered that the average fluctuations in the water levels did not follow a random sequence but linked to the length of the time intervals, over which he conducted his measurements [11].

Therefore, to standardize the measurement over time, Hurst divided the range by the standard deviation of the observed values, creating a dimensionless ratio known as the Hurst Exponent, and developed the *R/S* analysis method based on this [12]. The Hurst exponent offers a wide range of applications across various types of time series, due to its minimal limitations on the time series being studied. When estimating the Hurst exponent, there is no need to make any assumptions about the shape of the sequence distribution, which allows for the differentiation between random and non-random sequences.

The Hurst exponent (denoted as *H*) corresponds to three types of time series as follow,

First, when *H* = 0.5, the time series is a random walk. The value at different time in the sequence is random and independent, meaning there is no correlation between the data of the past and the future.

Second, when 0<H<0.5, the time series exhibits anti-persistence, demonstrating an expectation-reverting pattern. In other words, if a time series is on the rise in one period, it is likely to decline in the next. The past price increase suggests the future price decrease, and vice versa. The intensity of this anti-persistence is linked to how close *H* approaches 0. The more the value of *H* approaches 0, the more the time series resembles a random sequence.

Thirdly, when 0.5<H<1, the time series demonstrates long-term memory characteristics, indicating that past trends have a lasting influence on future behavior. In other words, if the past prices have risen, the future prices are likely to rise, and if the past prices have fallen, the future prices are likely to fall [13].

The *R/S* analysis method [12, 14], as a method to calculate the Hurst Exponent *H*, is shown as follows,

For a time series {*x*_*t*_} where *t* = *1, 2, ... t*, divided into blocks of size *n,* compute the mean for each block,

$${\bar{x}}_{n}=\frac{1}{n}\sum_{i=1}^{n}x_{i}$$ (1)

Calculate the range *R* of cumulative deviations from the mean,

$$R\left(n\right)=\max_{\left(1\leq k\leq n\right)}\sum_{i=1}^{k}\left(x_{i}-{\bar{x}}_{n}\right)-\min_{\left(1\leq k\leq n\right)}\sum_{j=1}^{k}\left(x_{j}-{\bar{x}}_{n}\right)$$ (2)

Determine the standard deviation *S*,

$$S\left(n\right)={\left[\frac{1}{n}\sum_{i=1}^{n}{\left(x_{i}-{\bar{x}}_{n}\right)}^{2}\right]}^{\frac{1}{2}}$$ (3)

Compute the R/S statistics,

$$Q_{n}=\frac{R\left(n\right)}{S\left(n\right)}$$ (4)

Then

$$Q_{n}=Cn^{H}$$ (5)

In Eq 5, *H* is the Hurst exponent, and *C* is a constant as n→∞, then linearize Eq 5 for estimation,

$$ln\left(Q_{n}\right)=lnC+Hln\left(n\right)$$ (6)

Then, the approximate value of *H* is,

$$H\approx \frac{lnQ_{n}}{ln\left(n\right)}$$ (7)

### 2.2. ARIMA model

Autoregressive Integrated Moving Average Model (ARIMA) is a generalized model of Autoregressive Moving Average (ARMA) that combines Autoregressive process and Moving Average processes and builds a composite model of the time series. As acronym indicates, ARIMA (*p, d, q*) captures the key elements of the model. Autoregression (AR): A regression model that uses the dependencies between an observation and a number of lagged observations (*p*). Integrated (I): To make the time series stationary by measuring the differences of observations at different time (*d*). Moving Average (MA): An approach that takes into accounts the dependency between observations and the residual error terms when a moving average model is used to the lagged observations (*q*) [15].

For the AR (*p’*) model, the forecasted value *p*_*t*_ is expressed as a linear combination of the past value *p’* of the sequence plus a constant *Con* and an error term $\varepsilon_{t}$. Then, the general form of the AR(*p’*) model is as follows,

$$p_{t}=\sum_{i=1}^{p\prime }\varphi_{i}p_{t-i}+Con+\varepsilon_{t}$$ (8)

Herein, *p*_*t*_ represents the value of the time series at time *t*; *Con* is a constant term; $\varphi_{i}$ are the autoregressive coefficients; $\varepsilon_{t}$, is the error term at time *t*, assumed to be white noise with the mean of 0 and the variance of $\sigma^{2}$.

For the MA(*q*) model, the forecast value *p*_*t*_ is expressed as a linear combination of the current value and *q* past values of the error term plus a constant. The general form of the MA(*q*) model is as follows,

$$p_{t}=\sum_{i=0}^{q}\theta_{i}\varepsilon_{t-i}+\mu$$ (9)

Herein, μ is the expectation of *p*_*t*_, $\theta_{\text{i}}$ are the moving average coefficients, and $\theta_{0}$ = 1. $\varepsilon_{t}$ is the error term at time *t*, assumed to be white noise with the mean of 0 and the variance of $\sigma^{2}$.

Integrate the AR*(p’)* and MA*(q)* to obtain the ARIMA(*p’,d, q*) model (assuming d=0) ,

$$p_{t}=\sum_{i=1}^{p\prime }\varphi_{i}p_{t-i}+Con+\varepsilon_{t}+\sum_{i=1}^{q}\theta_{i}\varepsilon_{t-i}$$ (10)

In this equation, $\varphi_{i}\neq 0$,$\theta_{\text{i}}\neq 0$,and $\sigma^{2}>0$. The parameters *p’* and *q* are referred to as AR and MA orders, respectively. ARIMA forecasting, also known as Box and Jenkins forecasting, is capable of handling non-stationary time series data due to its “integrate” step. Specifically, the “integrate” component involves differencing the time series to transform a non-stationary time series into a stationary one. The general form of an ARIMA model is denoted as ARIMA (*p’, d, q*) [16].

### 2.3. RNN and LSTM

**Fig 1:**
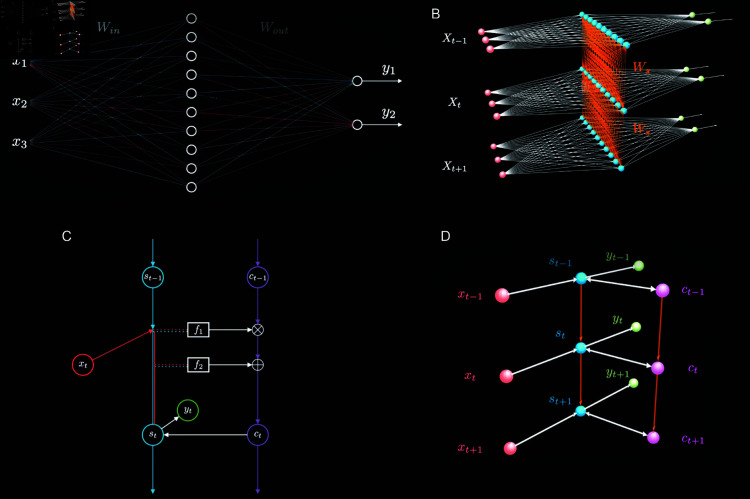
Neural network principle. Note: (a) illustrates the Simple Neural Network Principle; (b) illustrates the RNN Principle; (c) and (d) illustrate the LSTM Principle.

Fig 1a illustrates the foundational principle of a simple neural network. As shown in Fig 1a, a simple neural network consists of three main layers, namely, an input layer, a hidden layer and an output layer. The variables, $x_{\text{1}}$, $x_{\text{2}}$ and $x_{\text{3}}$, represent the data input into the input layer; the circles represent the neurons in the hidden layer; the variables, $y_{\text{1}}$ and $y_{\text{2}}$, represent the results output from the output layer. *W*_*in*_ represents the weight parameters from the input layer to the hidden layer, and *W*_*out*_ represents the weight parameters from the hidden layer to the output layer [17, 18].

If the output of a neuron is denoted as *s*_*i*_, there is the following equation,

$$s_{i}=f\left(\sum_{n}^{N}\left(w_{in}^{i}*x_{n}^{i}+b_{i}\right)\right)$$ (11)

Eq 11 clearly shows the sum over all inputs for neuron *i*, weighted by their respective weights, plus the bias, all passed through the activation function.

To generalize this for multiple neurons, matrix notation is used. The weight matrix *W* represents the weights for all neurons, the input vector *x* represents the inputs for all neurons, and the bias vector *b* represents the biases for all neurons. Then, Eq 11 then becomes Eq 12,

$$S=f\left(W_{in}X+b\right)$$ (12)

Here, *S* denotes the vector of neuron outputs, *W* represents the weight matrix, *X* is the input vector, *b* is the bias vector, and *f* is the activation function applied element-wise.

Deep neural networks, such as Convolutional Neural Networks (CNNs), usually extend horizontally, neglecting the changes of individual hidden layers over time. RNNs, on the contrary, can capture the continuous changing progress of each neuron in the hidden layer in the time dimension.

As shown in Fig 1b, in the case where the structure of the hidden layer maintains unchanged, its state repeats along the time axis, establishing temporal associations. The hierarchical expansion shown in Fig 1b is not a true increase in the number of neurons, but rather the state of the hidden layer at different time points. *W*_*s*_ represents the weight matrix between different layers. The RNN typically assumes that the *W*_*s*_ in different layers or at different time points, should be the same, so as to effectively reduce training parameters.

For the RNN, Eq 13 is derived by modifying Eq 12.

$$S_{t}=f\left(W_{in}X+W_{s}S_{t-1}+b\right)$$ (13)

It can be seen that compared to Eq 12, Eq 13 has an additional term $W_{s}S_{\text{t-1}}$, establishing an iterative relationship between the hidden layers at the different time points, giving neural network a memory capability.

RNNs achieve the memory capability by setting connections between the hidden layers at different time points. These connections allow the network to memorize previous information. However, it is only based on the previous time step, and it is a kind of short-term memory. Compared to RNNs, LSTMs add a new time chain that records long-term memory, enhancing the relationship between short-term memory and long-term memory, the process of which is shown in Fig 1c.

As shown in Fig 1c, the variables, $x_{\text{t-1}}$, *x*_*t*_, $x_{\text{t+1}}$ represent the input data at different time points; $s_{\text{t-1}}$, *s*_*t*_, $s_{\text{t+1}}$ and $c_{\text{t-1}}$, *c*_*t*_, $c_{\text{t+1}}$ represent the hidden layers, with $s_{\text{t-1}}$, *s*_*t*_, $s_{\text{t+1}}$ representing the short-term memory, and $c_{\text{t-1}}$, *c*_*t*_, $c_{\text{t+1}}$ representing the long-term memory; $y_{\text{t-1}}$, *y*_*t*_, $y_{\text{t+1}}$ represent the output data at different time points. Compared to RNNs, LSTMs add a time chain for long-term memory, enhancing the connections with chain of short-term memory. Taking time point *t* as an example, when calculating the hidden state *s*_*t*_, the data involved in the calculation includes the current input data *x*_*t*_ and the previous information that mainly involves the short-term memory $s_{\text{t-1}}$, and the long-term memory *c*_*t*_.

Fig 1d illustrates the operational principle of LSTMs in more details.

The forget gate ($f_{\text{1}}$) is defined by Eq 14,

$$f_{1}=sigmoid\left(w_{1}\left[\begin{array}{c} s_{t-1}\\ x_{t} \end{array}\right]+b_{1}\right)$$ (14)

The input gate (${\text{f}}_{2}$) is defined by Equation (15),

$$f_{2}=sigmoid\left(w_{2}\left[\begin{array}{c} s_{t-1}\\ x_{t} \end{array}\right]+b_{2}\right)*tanh\left(\hat{w_{2}}\left[\begin{array}{c} s_{t-1}\\ x_{t} \end{array}\right]+\hat{b_{2}}\right)$$ (15)

Subsequently, by integrating Eq 14 with Eq 15, we derive Eq 16 as shown below:

$$c_{t}=f_{1}*c_{t-1}+f_{2}$$ (16)

Herein, *c*_*t*_ is the memory cell state at the current time step, $c_{\text{t-1}}$ is the memory cell state from the previous time step, the *c*_*t*_ in Eq 16 will continue to propagate in the chain of long-term memory and update the current short-term memory *s*_*t*_.

### 2.4. RNN and LSTM for stock price forecasting

RNNs have been widely utilized for stock price forecasting. Moreover, it is discovered that compared to other deep learning models, RNNs exhibit significant superiority. Pawar, Jalem and Tiwari used an RNN to make forecasting on stock prices of Apple Inc., and the forecasting accuracy exceeded 95%, with a loss close to 0.1% [19]. Jahan and Sajal used an RNN to process the time series data of stock prices and made forecasts on stock prices. They checked the forecasting accuracy by cross-validating the forecasted closing prices and the actual prices, and found that the mean of the absolute value of the percentage of error is below 2%, indicating a very strong relation between the actual prices and the forecasted prices [20]. Later, Nabipour *et al*. used nine machine learning methods including Decision Tree, Random Forest, Adaptive Boosting (Adaboost), eXtreme Gradient Boosting (XGBoost), Support Vector Classifier (SVC), Naïve Bayes, K-Nearest Neighbors (KNN), Logistic Regression and Artificial Neural Network (ANN)) and two deep learning methods including an RNN and an LSTM to forecast the stock prices of four market groups on the Tehran Stock Exchange including diversified financial, petroleum, non-metallic minerals, and basic metals. The results showed that the RNN and the LSTM models had significantly better forecasting performance than the others [21].

During recent years, LSTMs are gradually replacing RNNs, and becoming the mainstream models used for stock price forecasting in the field of neural networks. Achyut Ghosh et.al used an LSTM to forecast the Indian stock market, and found that the longer the time window set in LSTM, the higher the forecasting accuracy [22]. Seng Hansun and Julio Christian Young used the LSTM model to forecast the stock prices included in the LQ45 Finance Industry Index (BBCA, BBNI, BBRI, BBTN, BMRI, and BTPS) and compared the forecasting accuracy with that of the ARIMA, the SVR, and the RNN models. The researchers found that the LSTM model had the best forecasting performance for both BBCA and BMRI, but not for the others [23]. This conclusion was further confirmed by Samarawickrama and Fernando’s research. The researchers selected daily stock prices of the companies listed on the Colombo Stock Exchange (CSE) as research samples, and used three models, namely, Simple Recurrent Neural Network model (SRNN), Gated Recurrent Unit model (GRU), and Long Short-Term Memory model (LSTM) to forecasting stock prices, and then compared the forecasting accuracy these models. The results showed that the forecasting accuracy of the SRNN model was about 99%, performing the best among the three, while for the other two models, GRU had relatively higher forecasting errors than the other [24].

Besides, researchers are trying to combine RNNs or LSTMs with other models to improve forecasting accuracy. In this aspect, Rather, Agarwal and Sastry proposed a hybrid model to forecast stock returns. The hybrid model they developed consists of three models: the ARMA model, the Exponential Smoothing model and the RNN model. Eventually, they found that the hybrid model achieved better forecasting performance, superior to that the simple RNN model [25]. Another two researchers, Shui-Ling and Li employed the ARIMA-RNN model for stock price forecasting. They found that this model not only overcame the volatility issue of a simple RNN but also avoided the over-fitting problems of neural networks. Compared with the RNN and ARIMA models, the ARIMA-RNN model demonstrated superior forecasting performance [26]. Li, Song and Tao used a Multi-task RNN with High-order Markov Random Fields (MRFs) to forecast the movement of stock prices. They conducted a comprehensive empirical study on three popular China’s stock market indices, and found that this model outperformed the baseline method [27]. Similarly, Bukhari *et al*. combined the LSTM with the ARFIMA (autoregressive fractional integrated moving average) to form the AFRIMA-LSTM hybrid model. This model not only minimized the volatility problem but also overcame the over fitting problem of neural networks. The forecasting results validated the effectiveness of the AFRIMA-LSTM hybrid model, which improved the forecasting accuracy by around 80% on RMSE, in comparison with traditional forecasting counterparts [28]. Pawar, Jalem, and Tiwari compared the deep learning model, named RNN-LSTM, with traditional machine learning models including Regression, Support Vector Machine, Random Forest, Feed Forward Neural Network, and Backpropagation, and found that RNN-LSTM model produced more accurate forecasts [19]. Lu *et al*. combined LSTM with CNN to form the CNN-LSTM model, to further improve the accuracy of financial time series forecasting accuracy on top of LSTM [8]. Lin *et al*. used a hybrid model combining the LSTM with the CEEMDAN (Complete Ensemble Empirical Mode Decomposition with Adaptive Noise) to forecast Standard & Poor’s 500 index (S&P500) and China Securities 300 Index (CSI300), and the empirical results presented that forecasting outcome of CEEMDAN-LSTM was optimal for both the advanced and emerging stock markets [29, 30].

Indeed, many studies have successfully enhanced forecasting accuracy by integrating RNNs or LSTMs with other types of models, however, very few have examined the method of enhancing the forecasting accuracy from the perspective of separating the long-term memory from the short-term memory components within a sequence, especially when forecasting time series using neural networks. Consequently, this paper endeavors to bridge this gap.

## 3. Methodology

### 3.1. Separation of long-term memory from short-term memory

The AR model is represented by the following equation,

$$p_{t}=\sum_{i=1}^{k_{1}}\varphi_{i}p_{t-i}+\varepsilon_{t}+Con$$ (17)

where *p*_*t*_ represents the value of the time series at time *t*. *Con* is a constant term, which can be considered as the mean of the series. $\varphi_{i}$ represent the autoregressive coefficients that capture the influence of past values on the current value. *i* denotes the order of the AR model, indicating the number of past values used to forecast the current value. $\varepsilon_{t}$ is the error term.

Typically, the value of *i* does not exceed 5, as discussed in Section 1. This implies that the AR model only captures the influence of short-term memory on *p*_*t*_. By letting $f_{short}(p_{t-i})$ denote this process, we obtain the following equation,

$$f_{short}\left(p_{t-i}\right)=\sum_{i=1}^{k_{1}}\varphi_{i}p_{t-i}$$ (18)

Subsequently, Eq 17 is transformed into Eq 19,

$$p_{t}=f_{short}\left(p_{t-i}\right)+\varepsilon_{t}+Con$$ (19)

Assuming that the variable *p*_*t*_ inherently possesses both long-term and short-term memory, when a term that captures the impact of long-term memory on *p*_*t*_ is incorporated into Equation 19, Equation 20 is derived.

$$p_{t}=f_{short}\left(p_{t-i}\right)+g_{long}\left(p_{t-i}\right)+\varepsilon_{t}+Con$$ (20)

Here, $f_{short}(p_{t-i})$ represents the influence of short-term memory on *p*_*t*_, while $g_{long}(p_{t-i})$ signifies the influence of long-term memory on *p*_*t*_. Additionally, $\varepsilon_{t}$ represents the random walk component, specifically the unpredictable fluctuations.

By modifying Eq 19 and Eq 20, we obtain Eq 21 and Eq 22,

$$p_{t}-f_{short}\left(p_{t-i}\right)=\varepsilon_{t(21)}+Con$$ (21)

$$p_{t}-f_{short}\left(p_{t-i}\right)=g_{long}\left(p_{t-i}\right)+\varepsilon_{t(22)}+Con$$ (22)

where $\varepsilon_{t}$ in Eq 21 and Eq 22 denotes the forecasting errors associated with these two equations, respectively.

Assuming that $f_{short}(p_{t-i})$ and $g_{long}(p_{t-i})$ are independent of each other, meaning that the presence of $f_{short}(p_{t-i})$ does not affect $g_{long}(p_{t-i})$, and vice versa, Eq 23 can be derived by using Eq 22 to minus Eq 21.

$$\varepsilon_{t(21)}=g_{long}\left(p_{t-i}\right)+\varepsilon_{t(22)}$$ (23)

where $\varepsilon_{t(21)}$ in Eq 21 serves the same function as $\varepsilon_{t}$ in Eq 17 based on the assumption that $f_{short}(p_{t-i})=\sum \phi_{\text{i}}p_{\text{t}-\text{i}}$, and the $\varepsilon_{t}$ in Eq 17 represents the forecasting errors of the AR model and is assumed to be white noise. Whereas, the $\varepsilon_{t(21)}$ in Eq 21 is not considered white noise but rather a non-stationary sequence due to the presence of $g_{long}(p_{t-i})$. Consequently, $f_{short}(p_{t-i})=\sum \phi_{i}p_{t-i}$ dose not hold under the condition that $f_{short}(p_{t-i})$ is a system capable of analyzing short-term memory, but lacks the capacity to analyze long-term memory, which leads to forecasting errors being a non-stationary sequence.

On the other hand, RNNs, which demonstrate superior performance in forecasting financial time series, are particularly adept at analyzing short-term memory information, but unequipped to handle long-term memory information, and their forecasting errors do not need to conform to the white noise assumption, thereby aligning with the requirements of $f_{short}(p_{t-i})$.

Eq 24 illustrates the forecasting process of RNNs.

$$p_{Train}\overset{input}{\to }RNN\overset{Train}{\to }RNNModel\overset{Forecast}{\to }{\hat{p}}_{RNN,Train}and{\hat{p}}_{RNN,Test}$$ (24)

When utilizing an RNN for time series forecasting, the initial step is to input the training set data into the RNN model to obtain a trained model. Subsequently, this trained RNN model is employed to forecast both the training set and the test set data. Consequently, Eq 21 is transformed into Eq 25, Eq 26, and Eq 27.

$$p_{t}-{\hat{p}}_{RNN}=\varepsilon_{t,RNN}$$ (25)

$$p_{t,Train}-{\hat{p}}_{RNN,Train}=\varepsilon_{t,RNN,Train}$$ (26)

$$p_{t,Test}-{\hat{p}}_{RNN,Test}=\varepsilon_{t,RNN,Test}$$ (27)

Based on the above analysis, if $f_{short}(p_{t-i})$ is replaced by an RNN model, then $\varepsilon_{t(21)}=\varepsilon_{t,RNN}$. Consequently, Eq 23 is transformed into Eq 28.

$$\varepsilon_{t,RNN}=g_{long}\left(p_{t-i}\right)+\varepsilon_{t}$$ (28)

The forecasting errors of the RNN model ($\varepsilon_{t,RNN}$) arise from what the RNN model can’t explain, including the effect of long-term memory on *p*_*t*_ and the random walk in stock prices ($\varepsilon_{t}$).

Therefore, it is essential to identify a model capable of analyzing the long-term memory information, thereby enabling the separation of $g_{long}(p_{t-i})$ from $\varepsilon_{t}$.

LSTMs possess the capability to analyze not only long-term memory but also short-term memory information. Consequently, this paper selects an LSTM to separate $g_{long}(p_{t-i})$ from $\varepsilon_{t}$. The forecasting process of LSTMs is illustrated in Eq 29.

$$\varepsilon_{t,RNN}\overset{input}{\to }LSTM\overset{Train}{\to }LSTMModel\overset{Forecast}{\to }{\hat{\varepsilon }}_{LSTM,Train}and{\hat{\varepsilon }}_{LSTM,Test}$$ (29)

By substituting Eq 24 and Eq 29 into Eq 20, we derive Eq 30, Eq 31, and Eq 32.

$$p_{t}={\hat{p}}_{RNN}+{\hat{\varepsilon }}_{LSTM}+\varepsilon_{t}$$ (30)

$$p_{t,Train}={\hat{p}}_{RNN,Train}+{\hat{\varepsilon }}_{LSTM,Train}+\varepsilon_{t,Train}$$ (31)

$$p_{t,Test}={\hat{p}}_{RNN,Test}+{\hat{\varepsilon }}_{LSTM,Test}+\varepsilon_{t,Test}$$ (32)

In Eq 25, the RNN model is used to capture the short-term memory information, occupying the role of $f_{short}(p_{t-i})$ and the LSTM model to capture the long-term memory information, taking the place of $g_{long}(p_{t-i})$, aiming to accomplish the separation of short-term memory and long-term memory information.

### 3.2. ARMA-RNN-LSTM hybrid modeling

This section uitilizes the ARMA-RNN-LSTM hybrid modeling approach to evaluate whether Eq 30 can efficiently separate the short-term memory information from the long-term.

Utilizing Eq 30, two distinct sets of forecasting results, denoted as ${\hat{p}}_{RNN}$ and ${\hat{p}}_{RNN}\;+\;{\hat{\varepsilon }}_{LSTM}$, , are generated. These forecasts, together with the actual values *p*_*t*_ from the training set, are subsequently input into the LSTM model to generate the final forecasting outcomes. This integrated approach, detailed in Eq 33, is designated as the ARMA-RNN-LSTM hybrid model in this paper.

$$p_{t,Train}and{\hat{p}}_{RNN,Train}{\hat{p}}_{RNN,Train}+{\hat{\varepsilon }}_{LSTM,Train}\overset{input}{\to }LSTM\overset{Train}{\to }\;LSTMModel\overset{Forecast}{\to }{\hat{\varepsilon }}_{LSTM,Train}and{\hat{\varepsilon }}_{LSTM,Test}$$ (33)

If the hybrid model achieves a significantly higher forecasting accuracy compared to the individual models (either the RNN or the LSTM), it confirms the effectiveness of this approach.

Comparing the ARMA-RNN-LSTM Hybrid Model with the ARMA model, we found the following similarities. The ARMA model consists of two components: one is the AR and another is the MA. When using the ARMA model to forecast stock prices, the AR component utilizes historical price information to forecast future prices, while the MA component uses error terms to forecast future prices. Whereas, in the hybrid model constructed, the RNN model is responsible for analyzing the historical price information, and the LSTM model is responsible for analyzing the information in the error terms. In summary, the structure of the hybrid model proposed in this paper shares some similarities with the ARMA model.

Comparing the ARMA-RNN-LSTM hybrid model with the traditional ARMA models, several similarities can be observed. The ARMA model comprises two main components: the AR component and the MA component. In the context of forecasting stock prices, the AR component leverages historical price data to forecast future prices, while the MA component utilizes error terms to refine these forecasts.

In contrast, the hybrid model proposed in this paper integrates more advanced techniques. Specifically, the RNN component is designed to analyze the historical price information of stocks, capturing short-term dynamics. Meanwhile, the LSTM component focuses on analyzing the error terms, which often contain long-term dependencies and trends. This division of labor allows the hybrid model to address both short-term and long-term memory in the data.

In summary, while the hybrid model incorporates more sophisticated methods, its structure shares conceptual similarities with the ARMA model in terms of separating the analysis of historical prices and error terms.

## 4. Test

This paper selects the daily closing prices of the SSE, the SZSE and the HSI as sample sequences, and utilizes the Hurst exponent to identify the sequences with the long-term memory. The RNN model (${\hat{p}}_{RNN}$), the RNN + LSTM model ( ${\hat{p}}_{RNN}\;+\;{\hat{\varepsilon }}_{LSTM}$), the LSTM model (${\hat{p}}_{LSTM}$), as well as the ARMA-RNN-LSTM hybrid model are utilized to forecast these sequences.

To elaborate further, the daily closing prices of the SZSE are selected as the sample sequence for the experimental test. The Hurst exponent of this sequence indicates the presence of long-term memory characteristics within the selected time window, making it an appropriate sample sequence for the ARMA-RNN-LSTM hybrid model. The daily closing prices of the HSI are chosen as the sample sequence for the robustness test. Similar to the SZSE, the Hurst exponent of the HSI sequence reveals the presence of long-term memory characteristics within the selected time window. This allows us to assess the applicability of the ARMA-RNN-LSTM hybrid model across different market environments. The daily closing prices of the SSE are selected as the sample sequence for the heterogeneity test. The Hurst exponent of the SSE sequence indicates an absence of long-term memory within the selected sample window. According to theoretical analysis, the ARMA-RNN-LSTM hybrid model is not suitable for forecasting sequences that lack long-term memory.

### 4.1. Experimental test

This section utilizes the daily closing prices of the SZSE, collected from July 27th, 2022, to March 19th, 2024, encompassing a total of 400 data points, as a sample dataset. Specifically, the first 250 data points, ranging from July 27th, 2022, to August 4th, 2023, constitute the training set. The subsequent 150 data points, spanning from August 7th, 2023, to March 19th, 2024, constitute the test set. Additionally, the data of this stock index are sourced from Bloomberg. Python 3.1.2 is utilized for modelling and data visualization. Stata is employed for conducting data statistics.

**Fig 2:**
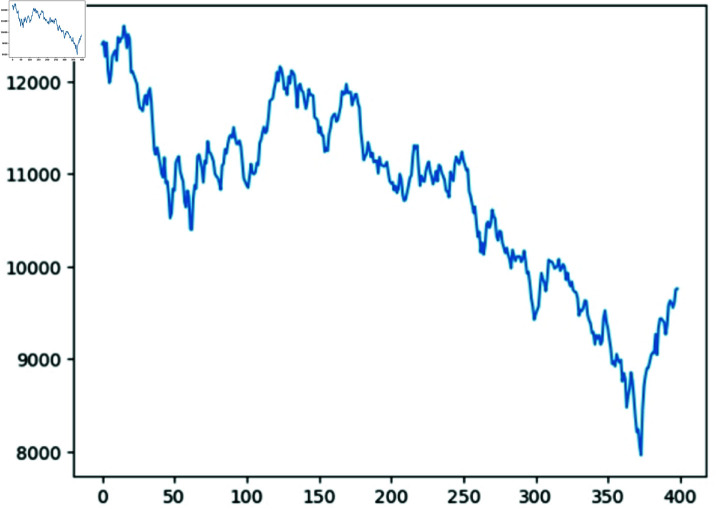
Daily closing prices of SZSE within selected time window

Fig 2 illustrates the fluctuations of the daily closing prices of the SZSE within the selected time window spanning from July 27th, 2022, to March 19th, 2024.

Additionally, the Hurst exponent for the sequence of the daily closing prices of the SZSE within selected time window stands at 0.63. This value, exceeding 0.5, indicates the presence of long-term memory and trend persistence in the sequence, suggesting that past price movements can influence the future trends. Table 1 provides the descriptive statistics for this dataset.

**Table 1:** Descriptive statistics for daily closing prices.

	Mean	SD	Min	Max
SZSE	10748.13	1004.51	7965.46	12595.00
HSI	18546.57	1686.29	14687.02	22688.90
SSE	3133.46	135.84	2702.00	3395.19

This paper employs the RNN model (${\hat{p}}_{RNN}$), the LSTM model (${\hat{p}}_{LSTM}$), the RNN+LSTM model (${\hat{p}}_{RNN}\;+\;{\hat{\varepsilon }}_{LSTM}$) , and the ARMA-RNN-LSTM hybrid model to forecast the daily closing prices of the SZSE within the selected time window, and conducts descriptive statistics on the forecasting errors, denoted as $\varepsilon_{t}$, generated during the forecasting process. Following this, the value of MAD (Median Absolute Deviation) and MAE (Mean Absolute Error) of $\varepsilon_{t}$ are utilized as criteria to assess the forecasting performance of each model, with the results presented in Table 2.

**Table 2:** Descriptive statistics for forecasting errors.

		${\hat{p}}_{RNN}$	${\hat{p}}_{LSTM}$	${\hat{p}}_{RNN}+{\hat{\varepsilon }}_{LSTM}$	ARMA-RNN-LSTM hybrid model
SZSE	Mean	-107.16	-236.73	-61.15	-13.15
	SD	141.23	217.92	121.00	30.60
	MAE	145.65	268.17	108.60	24.79
	MAD	128.99	244.90	92.53	19.48
	Min	-491.16	-859.27	-391.48	-121.85
	Max	358.96	480.80	230.05	101.58
HSI	Mean	62.88	-30.83	-2.82	-32.86
	SD	262.94	263.96	198.80	54.71
	MAE	217.85	207.73	162.71	41.89
	MAD	183.86	173.52	147.57	18.57
	Min	-509.36	-802.08	-495.57	-240.49
	Max	717.23	669.60	577.11	18.77
SSE	Mean	-55.73	-100.86	-61.71	-58.03
	SD	37.30	60.13	44.32	42.48
	MAE	56.21	101.50	62.96	60.96
	MAD	48.83	88.14	55.86	57.12
	Min	-48.83	-88.14	-55.86	-56.88
	Max	19.90	19.22	32.94	63.66

Table 2 demonstrates the forecasting accuracy of each model, ranked from the lowest to the highest, ${\hat{p}}_{LSTM}<{\hat{p}}_{RNN}<{\hat{p}}_{RNN}\;+\;{\hat{\varepsilon }}_{LSTM}<\text{ARMA-RNN-LSTM}$ hybrid model. Notably, the ARMA-RNN-LSTM hybrid model exhibits a significant improvement in forecasting accuracy, with an MAE of 24.79 and a MAD of 19.48.

Fig 5a reveals that the regression coefficients (R) of the ARMA-RNN-LSTM hybrid model are the highest compared to the other models. Furthermore, S1a Fig (Appendix 2) shows that the primary distribution of forecasting errors for the ARMA-RNN-LSTM hybrid model falls within the range of –100 to 100, which is the narrowest compared to other models. These observations indicate that the forecasting accuracy of the ARMA-RNN-LSTM hybrid model is significantly superior to that of other models, and this finding is consistent with the results presented in Table 2.

### 4.2. Robustness test

This section utilizes the daily closing prices of the HSI from July 27th, 2022 to March 5th, 2024, comprising a total of 400 data points, as the sample dataset. The first 250 data points, covering the period from July 27th, 2022 to July 26th, 2023, are used as the training set, and the remaining 150 data points, covering the period from July 26th, 2023, to March 5th, 2024, are used as the test set.

**Fig 3:**
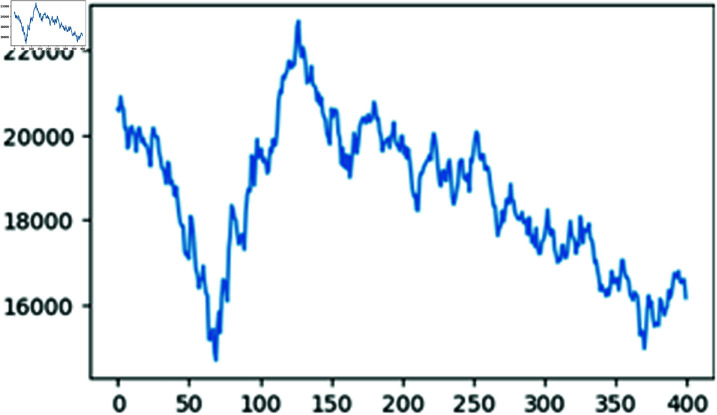
Daily closing prices of HSI within selected time window.

Fig 3 illustrates the fluctuations of the daily closing prices of the HSI within the selected time window spanning from July 27th, 2022, to March 5th, 2024.

Additionally, the Hurst exponent for the sequence of the daily closing prices of the HSI within the selected time window stands at 0.57. This value exceeds 0.5, indicating that the sequence exhibits long-term memory and suggesting that past price movements can influence future price trends. Additionally, Table 1 presents the descriptive statistics for this dataset, while Table 2 displays the descriptive statistics for the forecasting errors associated with the same dataset.

Table 2 presents the forecasting accuracy of each model, ranked from lowest to highest: ${\hat{p}}_{RNN}\approx {\hat{p}}_{LSTM}<{\hat{p}}_{RNN}\;+\;{\hat{\varepsilon }}_{LSTM}<\text{ARMA-RNN-LSTM}$ hybrid model. This ranking indicates that the ARMA-RNN-LSTM hybrid model exhibits the best forecasting performance compared to other models. This finding is consistent with those reflected in the SZSE.

Fig 5b indicates that the regression coefficient of the ARMA-RNN-LSTM hybrid model is the highest among the four models. S1b Fig (Appendix 2) reveals that the primary distribution of forecasting errors for the ARMA-RNN-LSTM hybrid model ranges from -200 to 0, which is the narrowest among all models. All these indicate that the forecasting accuracy of the ARMA-RNN-LSTM hybrid model is significantly superior to that of the other models, and this result is consistent with that in Table 2.

The result is consistent with that for the SZSE and passes the robustness test.

### 4.3. Heterogeneity test

**Fig 4:**
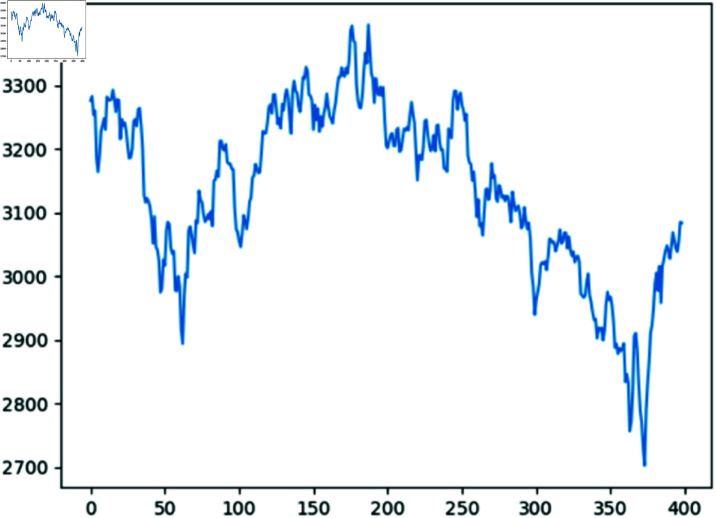
Daily closing prices of SSE within selected time window.

This section utilizes the daily closing prices of the SSE, collected from July 27th, 2022, to March 19th, 2024, encompassing a total of 400 data points, as a sample dataset, refer to Fig 4. Specifically, the first 250 data points, ranging from July 27th, 2022, to August 4th, 2023, constitute the training set. The subsequent 150 data points, spanning from August 7th, 2023, to March 19th, 2024, constitute the test set.

Fig 4 illustrates the fluctuations of the daily closing prices of the SSE within the selected time window spanning from July 27th, 2022, to March 19th, 2024.

Additionally, the Hurst exponent for the sequence of the daily closing prices of the SSE within the selected time window stands at 0.52. This value, which is approximately equal to 0.5, indicates that the sequence does not exhibit long-term memory, but only short-term memory. The descriptive statistics for the sequence are presented in Table 1.

Table 2 displays the forecasting accuracy of the models, ranked from the lowest to the highest: ${\hat{p}}_{LSTM}<\text{ARMA-RNN-LSTM}$ hybrid model$\approx {\hat{p}}_{RNN}\;+\;{\hat{\varepsilon }}_{LSTM}<{\hat{p}}_{RNN}$.

**Fig 5:**
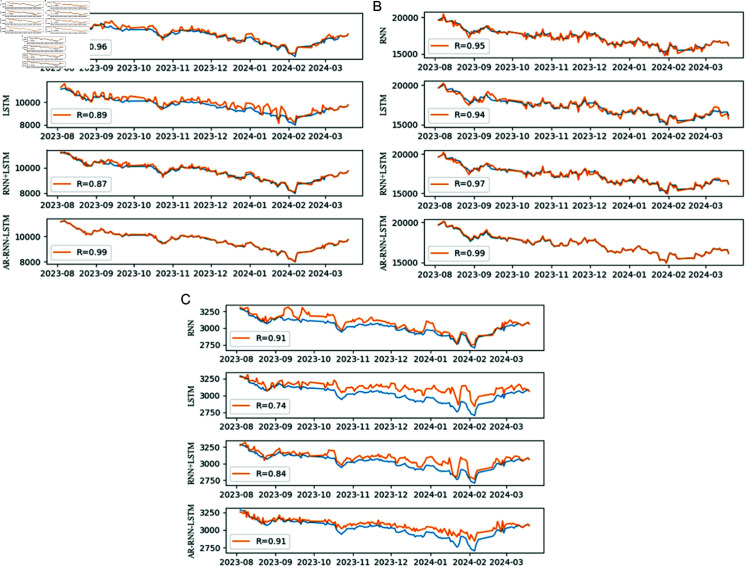
Model’s forecasting results. Note: ( **a**– **c**) forecasting results for the SZSE, the HSI, and the SSE, respectively. The blue lines represent the actual data trend, and the orange lines indicate the forecasted data trend; on the left side, RNN represents ${\hat{p}}_{RNN}$, LSTM represents ${\hat{p}}_{LSTM}$, RNN+LSTM represents ${\hat{p}}_{RNN}\;+\;{\hat{\varepsilon }}_{LSTM}$, and AR-RNN-LSTM represents the hybrid forecasting results. R represents the regression coefficient.

Fig 5c demonstrates the regression coefficients of the selected models, revealing that the RNN model’s coefficients align closely with those of the ARMA-RNN-LSTM hybrid model and are slightly elevated compared to other models. Additionally, Fig 5c indicates that there is insignificant difference in forecasting errors among these models. Furthermore, based on S1c Fig (Appendix 2), it is impossible to identify which model has the higher forecasting accuracy.

To summarize, the RNN model performs better in forecasting the SSE than the ARMA-RNN-LSTM hybrid model, indicating that for the sequence lacking long-term memory, the ARMA-RNN-LSTM hybrid model’s capacity to distinguish between the long-term memory from the short-term memory does not provide any advantages, resulting in no superior forecast performance.

## 5. Trading simulation

To illustrate the superiority of the proposed approach via a trading strategy and document its returns, this section designs two simulated trading scenarios aimed at evaluating the effectiveness of the ARMA-RNN-LSTM hybrid model in trading contexts.

The two scenarios are both designed on the assumption that the investors conduct equal-sized stock transactions in opposite directions at the start and end of each trading day. Specifically, if an investor bought stocks at the market open, they would sell an equivalent number of stocks before the market close, and vice versa. Otherwise, they should choose to be inactive for that day.

### 5.1. Scenario 1

For the first scenario, we consider specific stock markets, such as those in the United States, where investors have the option to either go long or short on stocks on a trading day. In such markets, if assuming that the intraday fluctuations adhere to a monotone function curve, then the maximum potential return can be calculated out.

$$W_{max}=\sum_{i=1}^{n}(|P_{future,i}-P_{future,i-1}|)$$ (34)

In Eq 34, *P*_*future*,*t*_ denotes the closing price of Day *t*; *n* represents the number of trading days within the time window, specifically, *n=150*; the absolute value of the difference that the opening price minus the closing price is regarded as the return of Day *t*; the sum of the returns over n trading days is regarded as the total return within the time window, denoted as *W*_*max*_. Since the dataset utilized in this paper exclusively closing prices, the closing price of Day *t*-*1* serves as a proxy for the opening price of Day *t*, which is based on the equivalence between these two prices without overnight trading behaviors.

Thus, the forecasting results for the selected models are classified into two categories: those that align with the actual trend, as exemplified in Eq 35, and those that diverge from the actual trend, demonstrated in Eq 36.

$$\left({\hat{P}}_{model,t}-P_{future,t-1}\right)*\left(P_{future,t}-P_{future,t-1}\right)>0$$ (35)

$$\left({\hat{P}}_{model,t}-P_{future,t-1}\right)*\left(P_{future,t}-P_{future,t-1}\right)<0$$ (36)

For the situation of Eq 35, it can be further subdivided into two specific cases, exemplified in Eq 37 and Eq 38 respectively.

$$\left|{\hat{P}}_{model,t}-P_{future,t-1}\right|>\left|P_{future,t}-P_{future,t-1}\right|$$ (37)

$$\left|{\hat{P}}_{model,t}-P_{future,t-1}\right|<\left|P_{future,t}-P_{future,t-1}\right|$$ (38)

Considering the situation outlined in Eq 37, and assuming that intraday fluctuation curves adhere to monotonic functions, the model’s forecasted price would remain unattainable even by the trading day’s end. Consequently, investors are unable to act on the model’s forecasts during trading hours, restricting their trading opportunities solely to the market close. Therefore, the investor’s actual return would be $w_{\text{1}}$.

$$w_{1,t}=\left|P_{future,t}-P_{future,t-1}\right|$$ (39)

Whereas, for the situation depicted by Eq 38, the stock price could attain the model’s forecasted levels prior to the market close. This enables investors to execute trades in anticipation, based on these forecasts. As a result, the investor’s realized return would be $w_{\text{2}}$.

$$w_{2,t}=\left|{\hat{P}}_{model,t}-P_{future,t-1}\right|$$ (40)

For the situation of Eq 36, if investing solely based on the model’s forecasts, investors would conduct trading actions opposed to the actual trend. In such case, the investor’s realized return would be $w_{\text{3}}$.

$$w_{3,t}=-\left|P_{future,t}-P_{future,t-1}\right|$$ (41)

Therefore, there is the following equation, which demonstrates the total return derived from the models’ forecasts in Scenario 1.

$$W_{model}=\sum_{i=1}^{n}\left(w_{1,i}+w_{2,i}+w_{3,i}\right)$$ (42)

Subsequently, to compare the returns derived from the selected models in the same dimension, this paper designs two ratios, denoted as $R_{\text{1}}$ and $R_{\text{2}}$.

$$R_{1}=\frac{W_{model}}{W_{max}}andR_{2}=\frac{W_{model}}{Cost}$$ (43)

In the Equations above, *cost* refers to the expenses incurred when purchasing stocks. Whereas, when calculating the returns of the models, it is assumed that investors are prohibited from reinvesting their earnings back into stocks.

The results demonstrated in Table 3 illustrate that, for the sequence that exhibits the long-term memory, such as the SZSE and the HSI, the returns derived from the ARMA-RNN-LSTM hybrid model forecasts are notably superior to those of other models, with $R_{\text{1}}$ of 81.65% and $R_{\text{2}}$ of 100.19% for the SZSE, and $R_{\text{1}}$ of 97.24% and $R_{\text{2}}$ of 144.92% for the HSI.

**Table 3:** Returns of models in Scenario 1.

		SZSE	HSI	SSE
$R_{\text{1}}$	$R_{\text{1,RNN}}$	61.93%	45.74%	44.78%
	$R_{\text{1,LSTM}}$	29.61%	64.74%	-17.36%
	$R_{\text{1,RNN+LSTM}}$	78.28%	58.41%	-11.22%
	$R_{\text{1,ARMA-RNN-LSTM}}$	81.65%	97.24%	-11.49%
$R_{\text{2}}$	$R_{\text{2,RNN}}$	75.99%	68.17%	48.73%
	$R_{\text{2,LSTM}}$	36.33%	96.49%	-18.89%
	$R_{\text{2,RNN+LSTM}}$	96.05%	87.05%	-12.21%
	$R_{\text{2,ARMA-RNN-LSTM}}$	100.19%	144.92%	-12.50%

However, in the case of the sequence lacking the long-term memory, such as the SSE, the ARMA-RNN-LSTM hybrid model’s forecasts fail to significantly enhance the returns, conversely, the RNN model’s forecasts achieve positive returns, with $R_{\text{1}}$ of 44.78% and $R_{\text{2}}$ of 48.73%.

### 5.2. Scenario 2

For the second scenario, we consider another type of stock markets, such as those in China, which allow investors to go long on stocks and prohibit short selling during a trading day. In such markets, under the assumption that intraday fluctuations follow a monotonic function curve, then the theoretical maximum return can be calculated as follows.

$${W^{\prime }}_{max}=\sum_{i=1}^{n}min\left[\left(P_{future,i}-P_{future,i-1}\right),0\right]$$ (44)

$W_{max}^{\prime }$ represents the theoretical maximum return achievable in the situation where investors engage in transactions only when the stock price rises, refraining from any activity during price declines.

Additionally, investors would initiate transactions when the model’s forecasted price during Day *t* (${\hat{P}}_{model,t}$) surpasses the actual closing price of Day *t-1* (${\hat{P}}_{\text{model,t-1}}$).

$${\hat{P}}_{model,t}-P_{future,t-1}>0$$ (45)

As such, two possible situations may arise: those aligning with the actual trend, as exemplified in Eq 46, and those diverging from the actual trend, demonstrated in Eq 47.

$$\left({\hat{P}}_{model,t}-P_{future,t-1}\right)*\left(P_{future,t}-P_{future,t-1}\right)>0$$ (46)

$$\left({\hat{P}}_{model,t}-P_{future,t-1}\right)*\left(P_{future,t}-P_{future,t-1}\right)<0$$ (47)

In the case of Eq 46, the return would be $w_{\text{1}}^{\prime }$

$${w^{\prime }}_{1,t}=min\left[{(P}_{future,t}-P_{future,t-1}),{(\hat{P}}_{model,t}-P_{future,t-1})\right]$$ (48)

In the case of Eq 47, the return would be $w_{\text{2}}^{\prime }$

$${w^{\prime }}_{2,t}=P_{future,t}-P_{future,t-1}$$ (49)

Finally, based on the preceding analysis, the return for a specific trading day can be articulated using Eq 50.

$$\begin{array}{c} {w^{\prime }}_{t}=\left\{min\left[{(P}_{future,t}-P_{future,t-1}),{(\hat{P}}_{model,t}-P_{future,t-1})\right]|{\hat{P}}_{model,t}-P_{future,t-1}>0\right\}\\ +\left\{0|{\hat{P}}_{model,t}-P_{future,t-1}<0\right\} \end{array}$$ (50)

In this case, the total return derived from the model’s forecasts can be calculated using Eq 51.

$$\begin{array}{c} {W^{\prime }}_{model}=\sum_{i=1}^{n}\\ \left\{min\left[{(P}_{future,t}-P_{future,t-1}),{(\hat{P}}_{model,t}-P_{future,t-1})\right]|{\hat{P}}_{model,t}-P_{future,t-1}>0\right\}\\ +\left\{0|{\hat{P}}_{model,t}-P_{future,t-1}<0\right\} \end{array}$$ (51)

In Eq 44, *cost* denotes the expenditure incurred when purchasing stocks. Whereas, when calculating the returns of the models, it is assumed that investors do not invest their earnings back into stocks.

Subsequently, to compare the returns derived from the selected models in the same dimension, this paper designs two ratios, denoted as $\text{R}{\text{'}}_{\text{1}}$ and $\text{R}{\text{'}}_{\text{2}}$.

$${R^{\prime }}_{1}=\frac{{W^{\prime }}_{model}}{{W^{\prime }}_{max}}and{R^{\prime }}_{2}=\frac{{W^{\prime }}_{model}}{Cost}$$ (52)

The results presented in Table 4 indicate that the highest returns for the HSI were achieved through decisions based on the forecasts of the ARMA-RNN-LSTM hybrid model, with $R_{\text{1}}^{\prime }$ of 96.96% and $R_{\text{2}}^{\prime }$ of 63.67%. The returns for the SZSE, also derived from the ARMA-RNN-LSTM hybrid model’s forecasts, are comparable to those from ${\hat{p}}_{RNN}\;+\;{\hat{\varepsilon }}_{LSTM}$, and superior to those of other models. The findings demonstrate that for the sequence exhibiting long-term memory, the forecasts of the ARMA-RNN-LSTM hybrid model is advantageous in enhancing the returns.

**Table 4:** Returns from different models in Scenario 2.

		SZSE	HSI	SSE
$R_{\text{1}}$	$R_{\text{1,RNN}}$	61.74%	44.20%	43.66%
	$R_{\text{1,LSTM}}$	25.36%	39.48%	-19.18%
	$R_{\text{1,RNN+LSTM}}$	83.22%	53.65%	-9.90%
	$R_{\text{1,ARMA-RNN-LSTM}}$	82.88%	96.96%	-6.22%
$R_{\text{2}}$	$R_{\text{2,RNN}}$	33.98%	29.02%	23.66%
	$R_{\text{2,LSTM}}$	13.95%	25.92%	-10.40%
	$R_{\text{2,RNN+LSTM}}$	45.80%	35.23%	-5.36%
	$R_{\text{2,ARMA-RNN-LSTM}}$	45.61%	63.67%	-3.37%

Conversely, for the SSE, the sequence lacking long-term memory, the ARMA-RNN-LSTM model’s forecasts never show any advantage into enhancing the returns. In such cases, investors can turn to standalone RNN models, as their forecasts can still furnish valuable information for devising investment strategies.

Finally, comparing the data presented in Table 3 and Table 4, it is evident that the ARMA-RNN-LSTM hybrid model demonstrates more advantages in forecasting stock prices that exhibit a downward trend than an upward trend.

In summary, the ARMA-RNN-LSTM hybrid method outperforms both the standalone RNN and LSTM in forecasting the sequence exhibiting long-term memory, thereby providing more valuable insights for enhancing the returns. However, for those lacking long-term memory, the ARMA-RNN-LSTM hybrid model’s forecasts hold limited significance. In such cases, investors may turn to the RNN model. All in all, the findings in this section reinforce the notion that the ARMA-RNN-LSTM hybrid model is more adept at handling the financial time series with long-term memory.

### 5.3. Scenario 3

For the third scenario, we deploy the hybrid model in real trading environment, exploring whether the model can assist investors to achieve excess profits in the real-world trading. The sample data selected in this section include three stock indices: two from Mainland China and one from Hong Kong, China, in which, short selling is prohibited, and only long selling is permitted for investors. The transaction mode utilized here is similar to that described in Scenario 2, where both the two markets adopt the *t+1* trading system, where stocks purchased on Day *t* can only be sold starting from Day *t+1* at the earliest, and it is assumed that investors purchase stock indices at the closing price on Day *t* and sell them at the closing price on Day *t+1*. It is worth noting this trading mechanism is implementable in the real world.

Meanwhile, we take into account of transaction costs in this scenario. For individual investors, it includes stamp duty, commission from securities companies, transfer fees or something else to be paid. The stamp tax should be charged upon selling stocks at a rate of 0.05%; commission fees should be charged upon both buying and selling stocks, with rates ranging from 0.15% to 0.3%, and transfer fees should also be charged upon both buying and selling at a rate of 0.001%. For institutional investors (securities companies), only stamp tax is required. This research assumes that the transaction cost rate for individual investors should be 0.5%, while that for institutional investors should be 0.05%.

Only when the forecasting results show an increase that is greater than transaction cost, would the trading occur. Therefore, if the costs are taken account for, Eq 38 needs to be modified to Eq 53,

$${\hat{P}}_{model,t}-P_{future,t-1}-TransactionCost>0$$ (53)

We believe that only when Eq 53 is satisfied, will investors choose to invest; otherwise, they will refrain from investing.

Taking these transaction costs into consideration, methods for calculating ${\text{W}}_{\text{max}}^{\prime }$ and ${\text{w}}_{\text{t}}^{\prime }$ need to be adjusted accordingly, as shown by following equations.

$${W^{\prime \prime }}_{max}=\sum_{i=1}^{n}min\left[W\prime max-TransactionCost,0\right]$$ (54)

$${w^{\prime \prime }}_{t}=min\left[{w^{\prime }}_{t}-TransactionCost,0\right]$$ (55)

Both the individual and the institutional investors would earn the corresponding returns based on the above analysis, which are summarized in Table 5 and Table 6, respectively.

**Table 5:** Returns earned by individual investors.

		SZSE	HSI	SSE
$R_{\text{1}}$	$R_{\text{1,RNN}}$	-52.03%	57.89%	-103.25%
	$R_{\text{1,LSTM}}$	-126.19%	49.09%	-173.67%
	$R_{\text{1,RNN+LSTM}}$	-3.21%	75.67%	-184.15%
	$R_{\text{1,ARMA-RNN-LSTM}}$	92.22%	93.52%	-131.46%
$R_{\text{2}}$	$R_{\text{2,RNN}}$	-15.47%	22.81%	-24.34%
	$R_{\text{2,LSTM}}$	-37.52%	19.34%	-40.94%
	$R_{\text{2,RNN+LSTM}}$	-0.95%	29.81%	-43.41%
	$R_{\text{2,ARMA-RNN-LSTM}}$	27.42%	36.84%	-30.99%

**Table 6:** Returns earned by institutional investors.

		SZSE	HSI	SSE
$R_{\text{1}}$	$R_{\text{1,RNN}}$	29.03%	72.71%	39.45%
	$R_{\text{1,LSTM}}$	5.76%	74.89%	24.08%
	$R_{\text{1,RNN+LSTM}}$	38.77%	79.33%	26.13%
	$R_{\text{1,ARMA-RNN-LSTM}}$	94.61%	95.99%	34.06%
$R_{\text{2}}$	$R_{\text{2,RNN}}$	14.77%	45.57%	19.91%
	$R_{\text{2,LSTM}}$	2.93%	46.93%	12.16%
	$R_{\text{2,RNN+LSTM}}$	19.73%	49.72%	13.19%
	$R_{\text{2,ARMA-RNN-LSTM}}$	48.15%	60.16%	17.20%

The data in Tables 5 and 6 demonstrate that both individual and institutional investors have achieved returns by making decisions based on ARMA-RNN-LSTM hybrid model’s forecasts for the SZSE and the HSI, which both exhibit long-term memory. These returns have reached more than 90% of the theoretical maximum profit (${\text{R}}_{1}>90\%$). Moreover, the hybrid model has enabled individual investors to attain returns at a rate of 30% and institutional investors at a rate of 50%. This underscores the effectiveness of the ARMA-RNN-LSTM hybrid model in actual trading scenarios, as the achieved returns have not only covered transaction costs but also generated more profits for both types of investors.

However, for stock indices lacking long-term memory properties, particularly the SSE, given that transaction costs are charged, the data in the tables indicate that no models are capable of assisting individual investors in realizing positive returns, while institutional investors have achieved profits at a rate between 10% to 20%. We attribute such a profit attainment primarily to their lower transaction costs compared to individual investors, rather than to the models’ forecasts.

### 5.4. Scenario 4

This section conducts an analysis on the returns achieved based on the hybrid model’s forecasts in both bull and bear markets.

Within the selected test set time window, the daily closing price fluctuations of both the SZSE and the HSI exhibit clear bull or bear market trends during some specific phases. In contrast, the fluctuations of the SSE do not exhibit any observable bull or bear market trends throughout the entire time window. Therefore, the focus of this analysis is directed towards the fluctuations of the SZSE and the HSI. Whatever, this Section also follows the market trading rules that allow investors to go long on stocks and prohibit short selling during a trading day.

**Fig 6:**
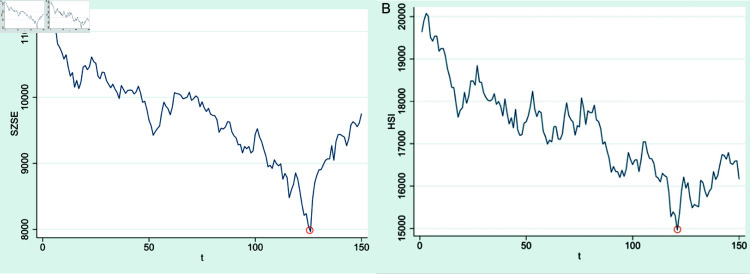
Daily closing prices of both SZSE and HSI within test set time window. Note: (1) ( **a**) SZSE and ( **b**) HSI; (2) The red circle in the figure marks the dividing line between bull and bear markets for the SZSE stock index price movements. The test set comprises the closing price data of 150 consecutive trading days, with the 116th trading day’s closing price highlighted in the red circle. The period from the first to the 116th trading day represents a bear market, whereas the period from the 116th to the 150th trading day signifies a bull market; (3) The red circle in the figure marks the dividing line between bull and bear markets for the HSI stock index price movements. The test set comprises the closing price data of 150 consecutive trading days, with the 121th trading day’s closing price highlighted in the red circle. The period from the first to the 121th trading day represents a bear market, whereas the period from the 121th to the 150th trading day signifies a bull market.

The daily closing price fluctuations of both the SZSE and the HSI throughout the entire test set time window is shown in Fig 6.

Assuming that investors’ decisions were exclusively based on the models’ forecasts, the potential returns they could achieve are detailed in Tables 7 and 8.

**Table 7:** Returns obtained from investment in SZSE.

		Bear Market		Bull Market	
		Total Returns	Average Daily Returns	Total Returns	Average Daily Returns
$R_{\text{1}}$	$R_{\text{1,RNN}}$	11.65%	0.10%	70.67%	2.02%
	$R_{\text{1,LSTM}}$	-21.02%	-0.18%	64.11%	1.83%
	$R_{\text{1,RNN+LSTM}}$	14.21%	0.12%	80.67%	2.30%
	$R_{\text{1,ARMA-RNN-LSTM}}$	90.85%	0.79%	98.84%	2.82%
$R_{\text{2}}$	$R_{\text{2,RNN}}$	3.63%	0.03%	15.98%	0.46%
	$R_{\text{2,LSTM}}$	-6.55%	-0.06%	14.50%	0.41%
	$R_{\text{2,RNN+LSTM}}$	4.43%	0.04%	18.24%	0.52%
	$R_{\text{2,ARMA-RNN-LSTM}}$	28.31%	0.25%	22.35%	0.64%

Note: Since the trading days of the bull market are different from those of the bear market, in order to more fairly compare the obtained returns in the bull market and the bear market, we calculate the average daily returns: the total return divided by the number of trading days.

**Table 8:** Returns obtained from investment in HSI.

		Bear Market		Bull Market	
		Total Returns	Average Daily Returns	Total Returns	Average Daily Returns
$R_{\text{1}}$	$R_{\text{1,RNN}}$	76.54%	0.64%	86.12%	2.87%
	$R_{\text{1,LSTM}}$	79.07%	0.66%	73.90%	2.46%
	$R_{\text{1,RNN+LSTM}}$	76.87%	0.64%	94.15%	3.14%
	$R_{\text{1,ARMA-RNN-LSTM}}$	98.57%	0.82%	93.24%	3.11%
$R_{\text{2}}$	$R_{\text{2,RNN}}$	36.60%	0.30%	15.37%	0.51%
	$R_{\text{2,LSTM}}$	37.81%	0.32%	13.19%	0.44%
	$R_{\text{2,RNN+LSTM}}$	36.76%	0.31%	16.80%	0.56%
	$R_{\text{2,ARMA-RNN-LSTM}}$	47.13%	0.39%	16.64%	0.55%

Note: Since the trading days of the bull market are different from those of the bear market, in order to more fairly compare the obtained returns in the bull market and the bear market, we calculate the average daily returns: the total return divided by the number of trading days.

As Table 7 shows, when relying on the ARMA-RNN-LSTM hybrid model’s forecasts, the average daily returns that the investors have achieved in the bear market is 0.25% ($R_{\text{2}}=0.25\%$), which is significantly higher than that from the other models. In contrast, in the bull market, the average daily returns that the investors have achieved is 0.64%, also higher than that from the other models but not as significantly as in the bear market.

The results presented in Table 8 is similar to those in Table 7. Specifically, under bear market conditions, the ARMA-RNN-LSTM hybrid model aids investors in achieving highest returns. However, under bull market conditions, the ARMA-RNN-LSTM hybrid model does not exhibit such significant advantages.

In summary, under bear market conditions, the ARMA-RNN-LSTM hybrid model can provide valuable guidance to investors, assisting them avoiding losses and even realizing profits. This also underscores the practical importance of the ARMA-RNN-LSTM hybrid model in formulating effective investment strategies in real-world trading scenarios.

## 6. Sensitivity analysis

This section adjusted the size of the training set and the test set, as well as the iteration count for the ARMA-RNN-LSTM hybrid model. Following adjustments to the training set size, 100 repeated experiments confirmed the model’s stable forecasting performance. Subsequent adjustments to the test set size and iteration count revealed that the ARMA-RNN-LSTM hybrid model maintains its forecasting edge over RNN and LSTM models for the time series with long-term memory information.

### 6.1. Size adjustment of training set

In Section 4, we selected 400 consecutive trading days as the time window, using the closing prices of these days as sample data. Specifically, the first 250 days’ data served as the training set, and the subsequent 150 days’ data served as the test set. Whereas, in this section, we modify the size of the training set by including the closing prices of 200, 300, 350, and 400 consecutive trading days, respectively, while maintaining the test set size unchanged, aimed at observing whether the results obtained after the adjustment are consistent with those presented in Section 4.

**Fig 7:**
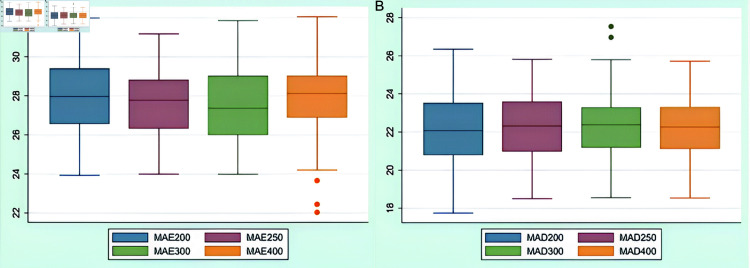
Results of forecasting SZSE. Note: In the figure, the numeral appended to “MAE” or “MAD” indicates the size of the training set. Specifically, MAE200 represents the MAE value generated in forecasting the SZSE using ARMA-RNN-LSTM hybrid model when the training set size is 200.

**Fig 8:**
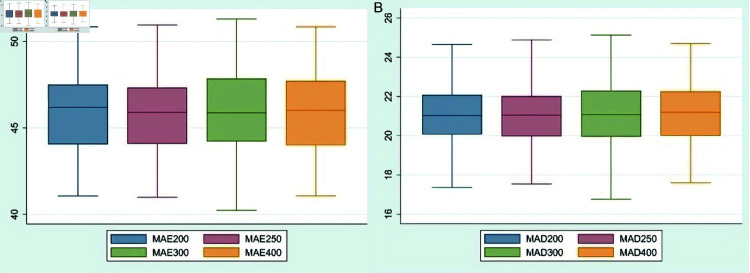
Results of forecasting HSI. Note: In the graph, the numeral appended to “MAE” or “MAD” indicates the size of training set. Specifically, MAE200 represents the MAE value generated in forecasting the HSI using ARMA-RNN-LSTM hybrid model when the training set size is 200.

Neural network models generate varying forecasting results each time they forecast the same sequence, with the forecasting errors displaying a certain degree of randomness. To effectively mitigate the impact of these random errors, this paper performs 100 rounds of repeated training and forecasting for each of the same-size training sets, followed by statistical analysis of the errors obtained from these rounds. Given that four training sets of different sizes are designed in this paper, a total of 400 forecasting rounds are conducted. The results of these analyses are depicted in Figs 7 and 8.

Figs 7 and 8 demonstrate that even when the window size of the training set is adjusted, there undergo no significant changes in MAE and MAD for the ARMA-RNN-LSTM hybrid model. This indicates that the ARMA-RNN-LSTM hybrid model is not sensitive to variations in the size of the training set data.

### 6.2. Size adjustment of test set

This section modifies the size of the test set, making it include the closing prices from 50 and 100 consecutive trading days, respectively, while maintaining a constant size for the training set, aimed at observing whether the results obtained after the adjustment are consistent with those presented in Section 4. The findings are presented in Tables 9 and 10.

**Table 9:** Results of forecasting SZSE.

	${\hat{p}}_{RNN}$	${\hat{p}}_{LSTM}$	${\hat{p}}_{RNN}+{\hat{\varepsilon }}_{LSTM}$	ARMA-RNN-LSTM hybrid model
MAE 50	131.94	337.23	99.10	24.88
MAD 50	126.76	346.54	94.77	20.85
MAE 100	136.5	289.88	98.13	27.08
MAD 100	127.34	269.93	91.22	21.38
MAE 150	145.65	268.17	108.6	24.79
MAD 150	128.99	244.90	92.53	19.48

Note: the numeral appended to “MAE” or “MAD” in the graph indicates the size of test set. Specifically, MAE50 represents the MAE value generated when forecasting the SZSE using the ARMA-RNN-LSTM hybrid model when the test set size is 50.

**Table 10:** Results of forecasting HSI.

	${\hat{p}}_{RNN}$	${\hat{p}}_{LSTM}$	${\hat{p}}_{RNN}\;+\;{\hat{\varepsilon }}_{LSTM}$	ARMA-RNN-LSTM hybrid model
MAE 50	242.94	175.45	176.94	10.13
MAD 50	213.10	165.84	168.87	10.28
MAE 100	226.45	172.94	167.00	20.12
MAD 100	187.87	136.13	154.89	12.60
MAE 150	217.85	207.73	162.71	41.89
MAD 150	183.86	173.52	147.57	18.57

Note: the numeral appended to “MAE” or “MAD” in the graph indicates the size of test set. Specifically, MAE50 represents the MAE value generated when forecasting the HSI using the ARMA-RNN-LSTM hybrid model when the test set size is 50.

The data presented in Tables 9 and 10 clearly show that the ARMA-RNN-LSTM hybrid model consistently outperforms other models in terms of MAE and MAD values, regardless of adjustments to the test set window size. This underscores the model’s ability to more accurately forecast the time series with long-term memory under the conditions of the different size of test set. These findings align with the results detailed in Section 4.

### 6.3. Adjustment of model iterations

Constructing the ARMA-RNN-LSTM hybrid model involves three types of components: RNN, LSTM, and LSTM, and each involves numerous parameters. This section centers on an important parameter, specifically, the number of iterations for model training. The aim is to explore whether the ARMA-RNN-LSTM hybrid model retains its advantage in forecasting accuracy for the time series with long-term memory after adjusting this parameter.

First, we adjust the number of iterations for the RNN model, the univariate LSTM model, and the multivariate LSTM model, respectively. We then use these adjusted models to forecast the SZSE and the HSI indices, with the results presented in Tables 11 and 12.

**Table 11:** Results of forecasting SZSE after adjusting iterations

	${\hat{p}}_{RNN}$	${\hat{p}}_{LSTM}$	${\hat{p}}_{RNN}\;+\;{\hat{\varepsilon }}_{LSTM}$	ARMA-RNN-LSTM hybrid model
${\text{RNN}}^{+}$	157.93	268.17	112.25	29.02
${\text{RNN}}^{-}$	139.66	268.17	106.49	24.42
${\text{LSTM (univariate)}}^{+}$	145.65	274.17	134.21	46.09
${\text{LSTM(univariate)}}^{-}$	145.65	255.63	120.54	31.41
LSTM (multivariate)+	145.65	268.17	108.60	35.60
LSTM(multivariate)-	145.65	268.17	108.60	33.84
Original model MAE	145.65	268.17	108.60	24.79

Note: “+” represents increasing the counts of iterations, and “-” represents decreasing the counts of iterations. For instance, “${\text{RNN}}^{+}$” means that in modeling process, only the iteration counts of the RNN increase, and those of the other models keep unchanged, with MAE values presented in the table. Due to the different initial iteration counts of the models, this paper adjusted the iterations by increasing or decreasing them by 10% based on their original counts.

**Table 12:** Results of forecasting HSI after adjusting iterations.

	${\hat{p}}_{RNN}$	${\hat{p}}_{LSTM}$	${\hat{p}}_{RNN}+{\hat{\varepsilon }}_{LSTM}$	ARMA-RNN-LSTM hybrid model
${\text{RNN}}^{+}$	209.04	207.73	162.53	42.47
${\text{RNN}}^{-}$	226.33	207.73	153.66	44.67
${\text{LSTM (univariate)}}^{+}$	217.85	209.84	155.57	38.3
${\text{LSTM(univariate)}}^{-}$	217.85	206.73	163.54	34.2
LSTM (multivariate)+	217.85	207.73	162.71	41.06
LSTM(multivariate)-	217.85	207.73	162.71	37.07
Original model MAE	217.85	207.73	162.71	41.89

Note: “+” represents increasing the counts of iterations, and “-” represents decreasing the counts of iterations. For instance, “${\text{RNN}}^{+}$” means that in modeling process, only the RNN increases iteration counts, and those of the other models keep unchanged, with MAE values presented in the table. Due to the different initial iteration counts of the models, this paper adjusts the iterations by increasing or decreasing them by 10% based on their original counts.

Tables 11 and 12 demonstrate that even after adjusting the number of iterations for the individual components of the ARMA-RNN-LSTM hybrid model, the forecasting results still show that the ARMA-RNN-LSTM hybrid model significantly outperforms the other models in terms of forecasting accuracy for both the SZSE and the HSI.

Subsequently, for other parameters, we conducted tests in a similar manner. The results indicate that the advantages of the ARMA-RNN-LSTM hybrid model in forecasting time series with long-term memory are universal, not affected by adjustments to the parameters of the model.

In summary, according to the experimental results presented in Section 6, adjustments to the size of the test set, as well as adjustments to the model’s parameters, have not led to any forecasts that contradicted the research hypotheses outlined in this paper. This suggests that the advantages of the ARMA-RNN-LSTM hybrid model in forecasting time series with long-term memory are universal, passing the sensitivity tests.

## 7. Discussion

### 7.1. Experimental result

LSTMs often exhibit superior forecasting accuracy compared to RNNs when handling time series data. This superiority stems from LSTMs’ unique gating mechanisms, which effectively tackle the vanishing and exploding gradient problems that frequently hinder RNNs. In RNNs, as information passes between time steps, the repeated multiplication of weights may lead to gradients vanishing or exploding, thus impairing the model’s ability to capture long-term dependencies. In contrast, LSTMs utilize their gating mechanisms to regulate information flow and maintain relevant information in lengthy sequences. This capability significantly enhances their forecasting accuracy, as supported by recent studies [31–33].

However, it is also worth noting that LSTMs do not always outperform RNNs in time series forecasting. For simpler time series or those with strong short-term dependencies, RNNs may actually perform better [34] than LSTMs. This observation underscores the inherent limitations of LSTMs.

Therefore, this paper proposes a method of ARMA-RNN-LSTM hybrid modelling, which combines the strength of RNNs and LSTMs. This method involves using an LSTM to re-forecast a specific time series that has already been forecasted by ${\hat{p}}_{RNN}\;+\;{\hat{\varepsilon }}_{LSTM}$ , aimed at yielding refined and improved forecasting results, and then, based on this method, we conduct an experiment, whose results show that for financial time series with long-term memory, the ARMA-RNN-LSTM hybrid model demonstrates significantly superior forecasting performance in comparison with both standalone LSTM and RNN models. This suggests that the component of ${\hat{p}}_{RNN}$ and ${\hat{\varepsilon }}_{LSTM}$ significantly contribute to separation of the long-term memory information from the short-term. On the other hand, the experimental results also show that for the financial time series lacking long-term memory, the forecasting performance of the ARMA-RNN-LSTM hybrid model is inferior to that of the RNN model, suggesting that the LSTM component in the hybrid model does not improve forecasting accuracy beyond the RNN model, but has an adverse effect. This phenomenon indicates that the LSTMs still undergo a process of separating the information of the long-term memory from the short-term during the training even if the forecasted time series lacking the long-term memory, thereby resulting in misclassification of certain data, and decreasing the model’s forecasting accuracy.

**Fig 9:**
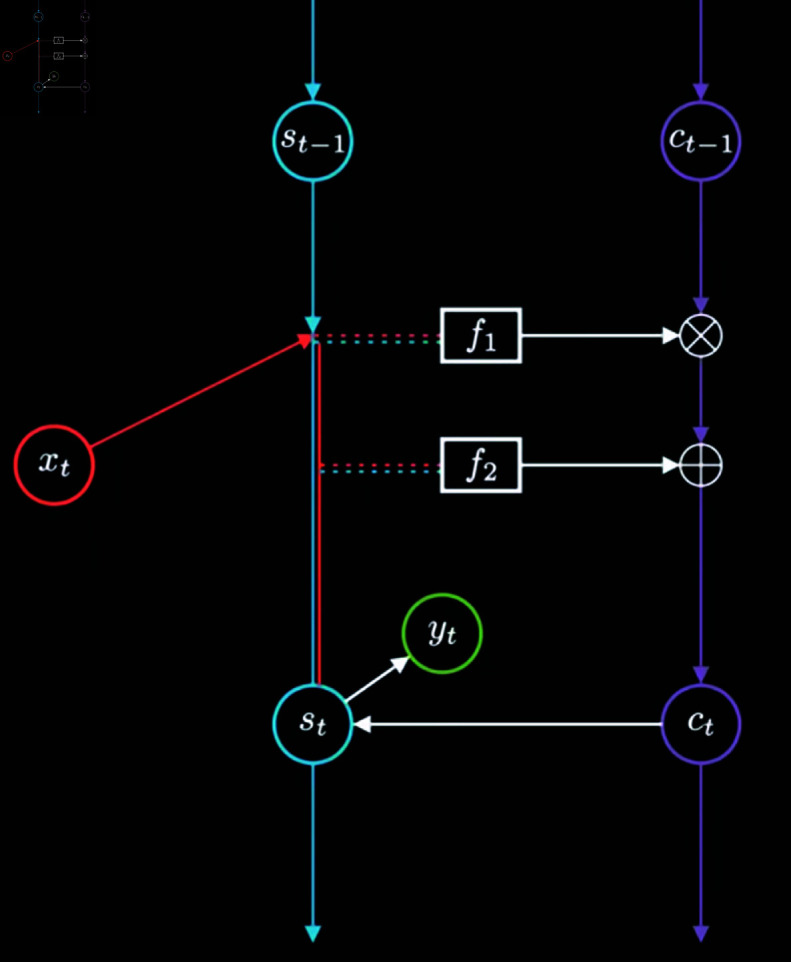
The principle of LSTM operation.

As seen in Fig 9, when using an LSTM to forecast the time series, the input data *x*_*t*_ is first separated into a short-term memory sequence *s*_*t*_ and a long-term memory sequence *c*_*t*_. Subsequently, the output result *y*_*t*_ is obtained based on both the long-term and short-term memory sequences [35]. Hypothetically, if we removed *c*_*t*_ from Fig 9, this forecasting process would then correspond to that of an RNN.

When respectively using an RNN and an LSTM to forecast the same time series with long-term memory, the results demonstrate that the LSTM’s forecasting accuracy is notably higher than that of the RNN. This is quite understandable given that LSTMs possess the capability to analyze long-term memory information, whereas RNNs lack this ability. However, when respectively using an RNN and an LSTM to analyze the same time series without long-term memory, and assuming that in this context the LSTM can perfectly separate the long-term memory information from the short-term within the sequence, theoretically, the LSTM’s forecasting accuracy should be similar to that of RNN. Yet, the actual results indicate that the forecasting accuracy of the RNN is significantly higher than that of the LSTM. This suggests that the LSTM has not yet fully distinguished two types of information within the sequence.

Consequently, we construct the ARMA-RNN-LSTM hybrid model, exploiting RNNs’ limitation that they can only analyze the short-term memory information in the sequence to isolate the short-term memory information from the sequence so that the two types of information could have been separated prior to the utilization of LSTMs, the experimental results show that such pre-separation is more effective than relying solely on LSTM’s internal mechanism to separate the long-term memory information form the short-term.

It is worth noting that previous research on forecasting errors generated by neural networks has predominantly focused on descriptive analysis or evaluated model forecasting accuracy using metrics such as MAE or MAD. The method proposed in this paper innovatively employs an LSTM to analyze these forecasting errors and subsequently validates the method’s feasibility through experimental data. This offers a fresh perspective for exploring the internal mechanisms of neural networks.

### 7.2. Information separation and leakage avoidance

As a matter of fact, the proposed ARMA-RNN-LSTM hybrid model seems to function as a signal decomposer when it handles the stock index series that contains long-term memory information, short-term memory information, and random walks, akin to EMD (Empirical Mode Decomposition) [36]: By exploiting the RNN’s limitation of solely analyzing short-term memory information, it effectively isolates the short-term memory components from the sequence. Additionally, it utilizes the LSTM’s potential of analyzing long-term information within the series to enable separation between long-term memory information and random walks. Ultimately, it employs an LSTM to handle the reintegrated sequence which distinctly features separated long-term and short-term memory information. Although the information separation via this hybrid model differs from the signal separation via EMD (refer to Appendix for the operating principle of EMD), the two types of separation both contribute to forecasting accuracy enhancement [37, 38].

Currently, many researches endeavor to improve time series forecasting performance by integrating neural network models with signal decomposers like EMD, EEMD, CEEMDAN, and ICEEMDAN, along with optimization algorithms: integrating Particle Swarm Optimization (PSO) with the signal decomposition technology to create the PSO-EMD method [39] and the PSO-EEMD method [40], integrating the Grey Wolf Optimizer (GWO) with the signal decomposition technology to create GWO-EEMD method [41] and the GWO-CEEMDAN method [42], and integrating the Whale Optimization Algorithm (WOA) with the signal decomposition technology to create the WOA-CEEMDAN method [43]. Furthermore, contemporary scholars have shifted their focus from optimizing parameters within signal decomposition technology using optimization algorithms to optimizing parameters in machine learning and deep learning models, leading to the development of the models such as the SWO-LSTM model, SSA-LSTM model, BWO-CONV-LSTM model [44, 45], PSO-KELM model, GWO-KELM model, WOA-KELM model [46–48], etc.

However, the issues identified in previous literature reveal that the use of mainstream signal decomposition models and deep learning methods often led to information leakage. Specifically, some of the information in the dataset intended for testing was unintentionally exposed to the model during training and testing, resulting in an inflated sense of forecasting accuracy on the test set. This phenomenon, known as information leakage, influences the performance of the model’s evaluation. The existence of information leakage leads to unreliable training and testing results, thereby preventing an accurate assessment of the model’s generalization capabilities.

Furthermore, in the context of time series forecasting, where historical data is used to forecast future trends, models must possess a certain level of generalizability. However, the existence of information leakage means that the information in the test set becomes known, causing the model to lose its potential to generalize to unknown data.

The impact of information leakage indeed has adverse effects on a model’s performance, causing the model to overfit to specific samples and patterns in the training set rather than learning generalized laws. This means that the model may perform well on the test set but not in real applications. If information leakage occurs, the evaluation of the model’s performance on the test set would be invalid for the test set data can not significantly represent unknown data. This may lead to a misleading understanding of the functionality and limitations of the model.

At present, scholars have recognized the problem of future unknown data involved in the decomposition process [49], and some scholars have adopted stepwise decomposition and other methods to prevent it [50]. However, excessive computational effort is still needed to improve the method, but so far no wide-scale research or methodological improvement has been made to address this issue [51].

The proposed method in this paper avoids information leakage that traditional signal decomposers struggle with. It separates data from the training set for training purpose and applies the trained model to forecast the test set data. This effectively avoids confusion between the training data and test set data.

Therefore, the ARMA-RNN-LSTM hybrid model, due to its embedded information separation mechanism, theoretically holds potential for partially replacing traditional signal decomposers. This modeling approach offers a encouraging direction for improving the performance of time series forecasting models.

However, future researches are needed to explore how to successfully incorporate this capability into diverse hybrid models and whether it can effectively improve the forecasting accuracy.

## 8. Conclusion and potential research directions

### 8.1. Conclusion

LSTMs exhibit distinct advantages over traditional time series models and other neural network framework when implementing financial time series forecasting. Their unique gating mechanisms, capabilities of managing long-term memory in sequences, nonlinear processing capabilities, scalability and flexibility make them powerful tools for this purpose.

However, for the time series with both the long-term and the short-term memory, an LSTM may not be an optimal choice, as their inherent mechanisms may not be able to adequately separate the long-term memory from the short-term memory information within a sequence, thereby containing their forecasting accuracy.

To overcome this limitation, this paper employs an RNN to capture the short-term memory information from historical price data, and concurrently utilizes an LSTM to capture the long-term memory information from the same dataset. The generated outputs from both the RNN and the LSTM are then fed into another LSTM for a second-round forecasting. This approach is termed as ARMA-RNN-LSTM hybrid modeling in this paper.

Subsequently, an experiment is made to validate the effectiveness of this modeling approach. Three time series are selected as sample sequences: the SSE, the SZSE, and the HSI. The Hurst exponent is employed as a tool to identify the sequence with long-term memory. The forecasting process is carried out using the RNN model, the RNN+LSTM model, the LSTM model, and the ARMA-RNN-LSTM hybrid model. Ultimately, the results reveal that for the time series with both short-term and long-term memory, the ARMA-RNN-LSTM hybrid model significantly outperforms both the LSTM and the RNN models. This superiority may arise from the ARMA-RNN-LSTM hybrid model’s more adequately separating the long-term memory information from the short-term within a sequence, which a standalone LSTM model can not achieve. Whereas, for the time series with only short-term memory, the RNN model performs better than the ARMA-RNN-LSTM hybrid model. This suggests that the LSTM component in this hybrid model may incorrectly classify some short-term memory information as long-term, thereby adversely affecting the overall forecasting accuracy. Therefore, we deduce that the ARMA-RNN-LSTM hybrid model significantly improves forecasting accuracy for time series with long-term memory, but not for those without. This improvement should be attributed to the hybrid model’s ability to effectively separate long-term memory information from short-term memory.

In conclusion, for the time series with long-term memory, the ARMA-RNN-LSTM hybrid model demonstrates superior forecasting accuracy compared to other models. Whereas, for the time series without long-term memory, the RNN model provides the best forecasting performance. Further, in trading contexts, for the series with long-term memory, the ARMA-RNN-LSTM hybrid model’s forecasts turn to yield higher returns compared to standalone RNN and LSTM. Conversely, for the series without long-term memory, the ARMA-RNN-LSTM hybrid model’s forecasts may not yield similar benefits, or even result in negative returns.

### 8.2. Potential research directions

This paper proposes an innovative approach that integrates econometric techniques with neural network methods to differentiate between long-term and short-term memory information within financial time series. This innovative method not only enhances the accuracy of financial price forecasting but also holds promise for boosting investment returns. However, it also opens up two directions for future research.

First, increasing the data frequency by converting daily price data into high-frequency trading data so that the time series lacking long-term memory characteristics could be converted into those that possess them. This idea is driven by the fact that the ARMA-RNN-LSTM hybrid model is fit for processing the series with long-term memory, and such a transformation in data frequency may convert the series without long term memory into those with such characteristics. However, it is important to note that this idea requires further verification through future research.

Second, transforming the ARMA-RNN-LSTM hybrid model from a univariate forecasting model to a multivariate forecasting model, thereby enabling its application in a broader range of fields. This idea is based on those that the efficient market hypothesis supports the use of historical stock prices for forecasting future prices, and acknowledges that multivariate forecasting models are more prevalent in such contexts. Nevertheless, this idea also warrants future investigation and validation.

## Supporting information

Appendix 1 Introduction of EMD. (DOCX)

Appendix 2 Forecasting error (εt). (DOCX)

Data Source The data of SZSE (CSV)

Data Source The data of HSI (CSV)

Data Source The data of SSE (CSV)
