# Supplementary material for: Enhanced separation of long-term memory from short-term memory on top of LSTM: Neural network-based stock index forecasting
Source: PLoS One. 2025 Jun 2;20(6):e0322737. doi: 10.1371/journal.pone.0322737 (PMC12129176; doi:10.1371/journal.pone.0322737)
Supplement: Appendix 1 — (DOCX) [file pone.0322737.s001.docx]

**Appendix 1 Introduction of EMD**

The Empirical Mode Decomposition (EMD) method is an adaptive and efficient technique used to decompose nonlinear and non-stationary signals [52,53]. It extracts a series of IMFs from the analyzed signal by sifting stage by stage. Each of these IMFs must satisfy the following conditions:

The first condition is that the number of extrema and the number of zero crossings must be equal or differ at most by one. The other point is that at any given time, the mean value of the envelope defined by the local maxima and the envelope defined by the local minima must be zero.

With the above definition for IMF, any signal can be decomposed in the following steps:

First step: Identify the local maxima and minima of the original data *x_t_*. Then connect them respectively with cubic spline lines to produce the upper and lower envelopes. Obtain the mean value of the corresponding data point *m_1_*, and define the difference between *x_t_* and *m_1_* as the first component *h_1_*.

$h_{1}=x_{t}-m_{t}$ (56)

Thus, the first sifting process is completed.

Second step: Taking *h_1_*as the original data *x_t_*, the second component *h_11_*can be obtained according to the operation described in the first step.

$h_{11}=h_{1}-m_{11}$ (57)

Repeat this step *k* times until *h_1k_* is an IMF,

$h_{1k}=h_{1(k-1)}-m_{1k}$ (58)

Designate it as *c_1_=h_1k_*, and select a stoppage criterion defined as follows:

$D_{k}=\frac{\sum_{t=0}^{T} \left| h_{1(k-1)}(t)-h_{1k}(t) \right|^{2}}{{\sum_{t=0}^{T} \left| h_{1(k-1)}(t) \right|}^{2}}$ (59)

Here, *D_k_* is smaller than a predetermined value.

Lastly, once *c_1_* is determined, the residual *r_1_* can also be obtained by separating *c_1_* from the rest of the data,

$r_{1}=x_{t}-c_{t}$ (60)

By taking the residual *r_1_* as new data and repeating the first and second steps, the second IMF component is obtained. If *c_1_* or *r_1_* is smaller than a predetermined value, or if *r_1_*becomes a monotone function, the sifting process is stopped; otherwise, it is repeated as the last step. Thus, a series of IMFs can be obtained.
