# Supplementary material for: Enhanced separation of long-term memory from short-term memory on top of LSTM: Neural network-based stock index forecasting
Source: PLoS One. 2025 Jun 2;20(6):e0322737. doi: 10.1371/journal.pone.0322737 (PMC12129176; doi:10.1371/journal.pone.0322737)
Supplement: Appendix 2 — (DOCX) [file pone.0322737.s002.docx]

**Appendix 2 Forecasting Error (*ε_t_*)**

(a)

(b)

(c)

**Figure S1.** Forecasting Error (*ε_t_*)

Note: Figures (a), (b), and (c) display the forecasting results for the SZSE, the HSI, and the SSE, respectively. From the top to the bottom, the sub-figure for the RNN represents the forecasting errors generated by the RNN model; the sub-figure for the LSTM represents the forecasting errors generated by the LSTM model; the sub-figure for RNN+LSTM represents the forecasting errors generated by $\hat{p}_{RNN}+\hat{\varepsilon}_{LSTM}$, and the sub-figure for the AR-RNN-LSTM denotes the forecasting errors generated by the AR-RNN-LSTM hybrid model.
